# Radiographic features of *Mycoplasma pneumoniae *pneumonia: differential diagnosis and performance timing

**DOI:** 10.1186/1471-2342-9-7

**Published:** 2009-04-29

**Authors:** Naoyuki Miyashita, Tadaaki Sugiu, Yasuhiro Kawai, Keiko Oda, Tetsuya Yamaguchi, Kazunobu Ouchi, Yoshihiro Kobashi, Mikio Oka

**Affiliations:** 1 Division of Respiratory Diseases, Department of Medicine, Kawasaki Medical School, Kurashiki, Okayama, Japan; 2 Department of Pediatrics, Kawasaki Medical School, Kurashiki, Okayama, Japan

## Abstract

**Background:**

The Japanese Respiratory Society guidelines propose a differential diagnosis for atypical pneumonia and bacterial pneumonia using a scoring system for the selection of appropriate antibiotic. In order to improve this scoring system, the guidelines are seeking new specific parameter. The purpose of this study was to clarify the pattern of abnormalities with *Mycoplasma pneumoniae *pneumonia on chest computed tomography (CT) and whether the radiographic findings could distinguish *M. pneumoniae *pneumonia from *Streptococcus pneumoniae *pneumonia.

**Methods:**

A retrospective review was performed of the CT findings of 64 cases and 68 cases where *M. pneumoniae *and *S. pneumoniae*, respectively, were the only pathogen identified by the panel of diagnostic tests used.

**Results:**

Of the 64 patients with *M. pneumoniae *pneumonia, bronchial wall thickening was observed most frequently (81%), followed by centrilobular nodules (78%), ground-glass attenuation (78%), and consolidation (61%). Bronchial wall thickening and centrilobular nodules were observed more often in *M. pneumoniae *patients than in *S. pneumoniae *patients (*p *< 0.0001). The presence of bilateral bronchial wall thickening or centrilobular nodules was only seen in patients with *M. pneumoniae *pneumonia. Using the scoring system of the Japanese Respiratory Society guidelines and chest CT findings, 97% of *M. pneumoniae *patients were suspected to be *M. pneumoniae *pneumonia without serology. When comparing the CT findings between early stage and progressed stage in the same patients with severe pneumonia, the radiographic features of early stage *M. pneumoniae *pneumonia were not observed clearly in the progressed stage.

**Conclusion:**

The present results indicate that the diagnosis of *M. pneumoniae *pneumonia would appear to be reliable when found with a combination of bronchial wall thickening and centrilobular nodules in the CT findings. However, these CT findings are not observed in progressed severe *M. pneumoniae *pneumonia patients.

## Background

Community-acquired pneumonia (CAP) is a common infection, which is sometimes fatal. Despite substantial progress in therapeutic options, CAP remains a significant cause of morbidity and death worldwide (the fourth leading cause of death in Japan). *Mycoplasma pneumoniae *is one of the most frequent causes of respiratory tract infections, accounting for as many as 10–30% of all cases of CAP [1-3]. Epidemiological studies in Japan have demonstrated that the incidence of *M. pneumoniae *pneumonia is the second leading pathogen of CAP next to *Streptococcus pneumoniae *[3,4]. Serological testing is currently the most common tool for the diagnosis of *M. pneumoniae *infection. However, this test requires paired serum samples with a two to four week interval and provides only a retrospective diagnosis.

The etiological matter that most clearly differentiates Asia from Western countries is the frequency of drug-resistant *S. pneumoniae*, and especially the high-level of macrolide-resistant *S. pneumoniae *[5-7]. Approximately half of *S. pneumoniae *cases show strong resistance to macrolides with minimum inhibitory concentrations greater than or equal to 128 μg/mL [5,7]. Therefore, it is difficult to manage both bacterial and atypical pneumonias by medication with macrolide antibiotics as the first-choice drug. In addition, in recent years the incidence of fluoroquinolone-resistant *S. pneumoniae *has risen markedly and particularly in patients over 60 years of age [7,8]. Furthermore, fluoroquinolone-resistant *Haemophilus influenzae *has also emerged [7,9]. Therefore, the usage of fluoroquinolones as an empirical first choice drug should be limited to only selected patients who have co-morbid illnesses, are of an older age group, have used antibiotics recently, or have severe CAP to prevent an increase in the frequency of quinoline-resistant strains [4].

These facts indicate that it could be important to distinguish atypical pneumonia, especially *M. pneumoniae *pneumonia, from *S. pneumoniae *pneumonia for the empirical selection of antibacterial therapy. Thus, the Japanese Respiratory Society (JRS) CAP guidelines are on trial to differentiate between atypical pneumonia and bacterial pneumonia using a scoring system [4]. The guidelines have six parameters and criteria based on clinical features of *M. pneumoniae *pneumonia [10]. However, using this system there are still many cases where the guidelines cannot differentiate between *M. pneumoniae *pneumonia and *S. pneumoniae *pneumonia, and the JRS guidelines are seeking new specific parameters in order to improve this scoring system [4]. Some reports have focused on radiographic features using high-resolution computed tomography (HRCT) of *M. pneumoniae *pneumonia [11-15]. However, to our knowledge, there have been relatively few comparative studies that have investigated the radiographic findings of *M. pneumoniae *pneumonia versus *S. pneumoniae *pneumonia. The purpose of this study was to clarify the pattern of abnormalities with *M. pneumoniae *pneumonia on chest CT and whether the radiographic findings could distinguish *M. pneumoniae *pneumonia from *S. pneumoniae *pneumonia. Furthermore, this study also evaluated the performance of CT scans take at different stages of infection.

## Methods

### Study population

The study was retrospective and included adult patients with CAP who underwent HRCT. In all patients, CT images were obtained before treatment with antibiotics. Patients were seen at Kurashiki Daiichi Hospital, Kawasaki Medical School Kawasaki Hospital, and Kawasaki Medical School Hospital, Okayama, Japan, between April 2000 and December 2008. None of the patients were immunocompromised; namely patients with HIV infection, neutropenia secondary to chemotherapy, or patients on immunosuppressants; or patients with history of chronic lung diseases, from nursing homes, or patients with recent (<30 days) admission to hospital. The diagnosis was based on clinical signs and symptoms (cough, fever, productive sputum, dyspnea, chest pain, or abnormal breath sounds), and radiographic pulmonary abnormalities that were at least segmental and were not due to pre-existing or other known causes. All cases of pneumonia occurring more than three days after hospitalization were considered nosocomial and were excluded. The study protocol was approved by the Ethics Committee at Kawasaki Medical School.

### Microbiological laboratory tests

Microbiological tests such as Gram stain, cultures, urinary antigen tests, and serological tests were performed as described previously [3,16]. Blood cultures and nasopharyngeal swab specimens were obtained from all patients on admission and, if pleural fluid and sputum were available, a Gram stain test and a quantitative culture were obtained. Sputum data were only evaluated when the Gram stain test revealed numerous leukocytes (>25 in a × 100 microscopic field) and few squamous epithelial cells (<10 in a × 100 microscopic field). Certain invasive methods such as bronchoscopic examination were employed to obtain specimens in some patients after full explanation of the procedures. These specimens were also used for culturing of *M. pneumoniae *and *Legionella *species on pleuropneumonia-like organism broth and agar (70% pleuropneumonia-like organism broth [Difco, Inc., Detroit, MI, USA] supplemented with 20% heat-inactivated horse serum, 10% fresh yeast extract [25%], thallium acetate [final concentration 0.5 mg/mL], and sterile penicillin G [final concentration 1,000 U/mL]) and buffered charcoal-yeast extract alpha agar, respectively. Cultures for *Chlamydophila pneumoniae *and *C. psittaci *were performed using cycloheximide-treated HEp-2 cells grown in a 24-well cell culture plate [16]. All specimens were passed twice. Culture confirmation was done by fluorescent-antibody staining with *C. pneumoniae *and *C. psittaci *species-specific and genus-specific monoclonal antibodies. Bronchoscopic specimens were also used for polymerase chain reaction (PCR) of *M. pneumoniae *and chlamydial species. The *M. pneumoniae-*specific primers used for the PCR were from the DNA base sequence within the P1 cytadhesin gene, and amplification was performed as reported by Ramirez et al. [17]. The *C. pneumoniae-*specific and genus-specific primers used for PCR were from the DNA base sequence within the 53-kDa protein gene and major outer membrane protein gene, respectively, established in our laboratory. These assays were performed as described previously [3,18].

Paired serum samples were collected at intervals of at least four weeks after onset. Complement fixation tests were done in all patients for antibodies to influenza A and B viruses, adenovirus, respiratory syncytial virus, cytomegalovirus, and parainfluenza virus types 1, 2, and 3. Antibodies to *M. pneumoniae *were measured by a passive agglutination test (Serodia-Myco II kit, Fujirebio, Tokyo, Japan), *Legionella *species by a microagglutination test (detection of *L. pneumophila *serogroups 1~6, *L. bozoemanii*, *L. dumoffii*, *L. gormanii*, and *L. micdadei*), and *Coxiella burnetii *by an indirect immunofluorescence test. A microimmunofluorescence test was used for the titration of IgG and IgM antibodies against chlamydial species, using formalinized elementary bodies of *C. pneumoniae *KKpn-15, *C. trachomatis *L2/434/Bu, and *C. psittaci *Budgerigar-1 strains as antigens [16]. Rheumatoid factors were absorbed with GullSORB (Meridian Bioscience Inc., OH, USA) before IgM titrations. In addition to serology, culturing, and/or PCR, a urinary antigen test (Binax NOW, Binax Inc. Portland, ME, USA) was used for the detection of *S. pneumoniae *and *L. pneumophila*.

### Criteria for the determination of microbial etiology

Microbial etiology was classified as "definitive", "presumptive", or "unknown" as reported previously [3]. Bacteria were considered to be definitive causative agents when isolated from blood or pleural fluid cultures. The results of sputum cultures were considered in combination with Gram stain findings. An organism showing heavy (≥ 10^7 ^cfu/mL) or moderate (10^6 ^cfu/mL) growth of a predominant bacterium on a sputum culture was considered to be a presumptive pathogen. Any microorganism isolated from bronchoscopic specimens was considered to be a presumptive pathogen when its concentration reached >10^5 ^cfu/mL in quantitative culture. If *M. pneumoniae *or *Legionella *species were isolated from a specimen, that specimen was considered to be a definitive pathogen even if the culture showed little growth. *L. pneumophila *and *S. pneumoniae *were considered to be presumptive agents when the urinary antigen test was positive. For serological tests, a four-fold rise in the antibody titer level between paired sera was considered definitive. Acute *C. pneumoniae *or *C. psittaci *infection was defined as IgM ≥ 1:32 or a four-fold rise in IgG or IgM titer between acute and convalescent serum samples.

### Chest CT findings

CT examinations were performed using a Hi-Speed Advantage scanner (General Electric Medical Systems, Milwaukee, WI, USA), Hi-Speed LX/i Advantage (General Electric Medical Systems), or a Light Speed Ultra (General Electric Medical Systems). All scans were obtained during suspended end inspiration with the patient in a spine position. HRCT was performed with 1-mm collimation at 10-mm intervals. Images were obtained at lung (level -700 HU; width, 1,500 HU) and mediastinal (level 20–40 HU; width, 400 HU) levels. The time between clinical onset of pneumonia (fever and/or respiratory symptoms) and CT ranged from 1 to 10 days (mean, 5.1 days) for *M. pneumoniae *pneumonia and from 1 to 12 days (mean, 5.2 days) for *S. pneumoniae *pneumonia. Contrast material was used in 12 patients with *M. pneumoniae *or *S. pneumoniae *pneumonia.

Three chest radiologists (16, 12 and 10 years experience, respectively), who were blinded to the diagnoses, retrospectively and independently assessed the presence of consolidation, ground-glass attenuation, centrilobular nodules, thickening of the bronchial wall, reticular or linear opacity, pleural effusion, and lymphadenopathy. Readers also evaluated if the pneumonia was unilateral or bilateral and identified the opacity pattern of pneumonia. In addition, it was recorded which lobe of the lung was involved.

Consolidation was defined as air-space opacification with obscuration of the underlying vasculature. Ground-glass attenuation was defined as mildly increased attenuation without obscuration of the underlying vasculature. A centrilobular nodule was defined as a nodule identified around the peripheral pulmonary arterial branches or 3 to 5 mm away from the pleura, interlobular septa, or pulmonary veins. Bronchial wall thickening was defined as the thickening identified widespread areas not close to areas of ground-glass attenuation and/or consolidation. Interlobular septa thickening, intralobular interstitial thickening, and areas of irregular linear opacity were all classified as reticular or linear opacity. The reticular framework in ground-glass attenuation that is described as crazy-paving appearance was not classified as an area of reticular or linear opacity. Mediastinal lymphadenopathy was judged to be present when the minimal diameter of a lymph node was larger than 10 mm. Hilar lymphadenopathy was judged to be present only if the maximum diameter of the ipsilateral hilum exceeded that of the contralateral hilum by 1.5-fold or more. The final decisions for the presence of each finding and the opacity pattern for each case were reached by the consensus of the three radiologists.

### Differential diagnosis in JRS guidelines

The JRS guidelines include six parameters and criteria based on underlying conditions, clinical symptoms, and laboratory findings by multiple regression analysis [10]. These parameters are; 1) under 60 years of age, 2) no or minor co-morbid illness, 3) the patient has stubborn cough, 4) the patient has poor chest auscultatory findings, 5) no sputum or identified etiological agent by rapid diagnostic tests, and 6) a peripheral white blood cell (WBC) count below 10,000/mm^3^. When there is a correlation of more than four parameters out of six, then the guidelines recommend the use of macrolides or tetracyclines for suspected *M. pneumoniae *pneumonia [4]. If these criteria are not met, the guidelines recommend the use of β-lactams for suspected *S. pneumoniae *pneumonia [4].

### Statistical analysis

Statistical analysis was performed using Stat View version 5.0. (SAS Institute Inc, Cary, NC, USA). The incidence of underlying conditions, clinical findings, and radiographic findings were analyzed using Fisher's Exact test. Mean age of patients and laboratory data were compared using Student's *t *test. For each radiological finding, kappa values were calculated between the readers.

## Results

Five hundred and sixty-five patients were enrolled in this study. A microbiological diagnosis was established in 298 patients. Among all CAP cases, there were 64 cases and 68 cases where *M. pneumoniae *and *S. pneumoniae*, respectively, was the only pathogen identified by the panel of diagnostic tests used. Cases of pneumonia mixed with other microorganisms were excluded in this study. All *M. pneumoniae *cases demonstrated four-fold antibody seroconversion (10 cases were culture positive).

Table 1 shows the clinical characteristics of pneumonia patients on admission to hospital. The mean age of the patients with *M. pneumoniae *pneumonia was significantly lower than that of those with *S. pneumoniae *pneumonia (*p *< 0.0001). The frequency of a co-morbid illness with *M. pneumoniae *pneumonia was significantly lower than with *S. pneumoniae *pneumonia (*p *< 0.0001). The most common co-morbid illness was diabetes mellitus (*M. pneumoniae *6, *S. pneumoniae *10), followed by cardiovasucular diseases (*M. pneumoniae *3, *S. pneumoniae *7), chronic liver disease (*M. pneumoniae *3, *S. pneumoniae *6), and cerebrovascular diseases (*M. pneumoniae *1, *S. pneumoniae *5). The mean WBC count on admission was significantly lower in patients with *S. pneumoniae *pneumonia (*p *< 0.0001). These results were almost identical to the findings of previous studies [16,19].

**Table 1:** Clinical characteristics in patients with *Mycoplasma pneumoniae *pneumonia and *Streptococcus pneumoniae *pneumonia on admission*

Characteristics	*M. pneumoniae*	*S. pneumoniae*	*p *value
Number	64	68	
Mean age (range), years	36.6 (18–69)	61.8 (19–86)	<0.0001
Male: Female	34: 30	42: 26	0.3791
Co-morbid illness	15 (23)	33 (48)	<0.0001
PSI risk classes**			
I – III	41 (64)	29 (43)	0.0154
IV	23 (36)	39 (57)	
V	0	0	
WBC mean (/μL)	7,400	13,800	<0.0001

*Data represent the numbers of patients, and numbers in parentheses are percentages.

**PSI, pneumonia severity index.

The kappa value between the readers was 0.607 for consolidation, 0.552 for ground-glass attenuation, 0.746 for centrilobular nodules, 0.564 for thickening of the bronchial wall, 0.587 for reticular or linear opacity, 0.882 for pleural effusion, and 0.618 for lymphadenopathy. These values indicated fair to good interreader agreement.

**Figure 1:**
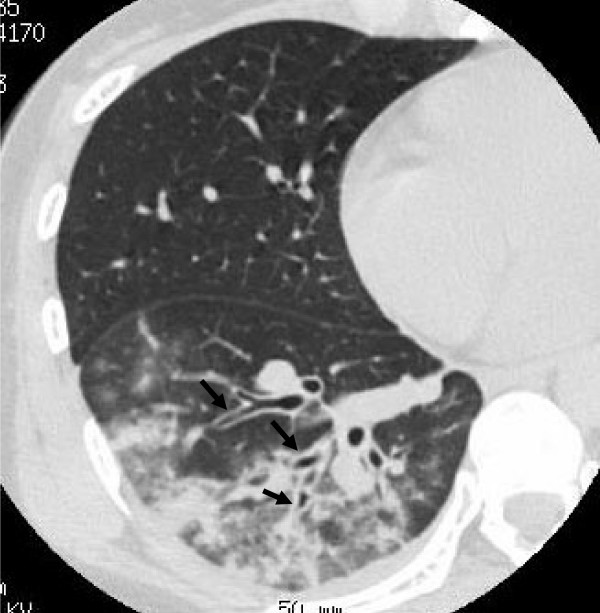
**30-year-old woman with *M. pneumoniae *pneumonia**. CT shows bronchial wall thickening (arrows). Lobular areas of consolidation and ground-glass attenuation are also seen.

**Figure 2:**
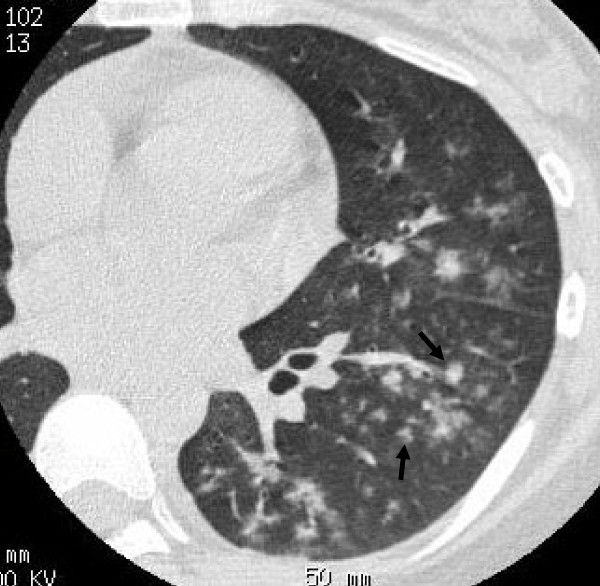
**24-year-old man with *M. pneumoniae *pneumonia**. CT shows centrilobular nodules (tree-in-bud appearance, arrows). Bronchial wall thickening is also seen.

**Figure 3:**
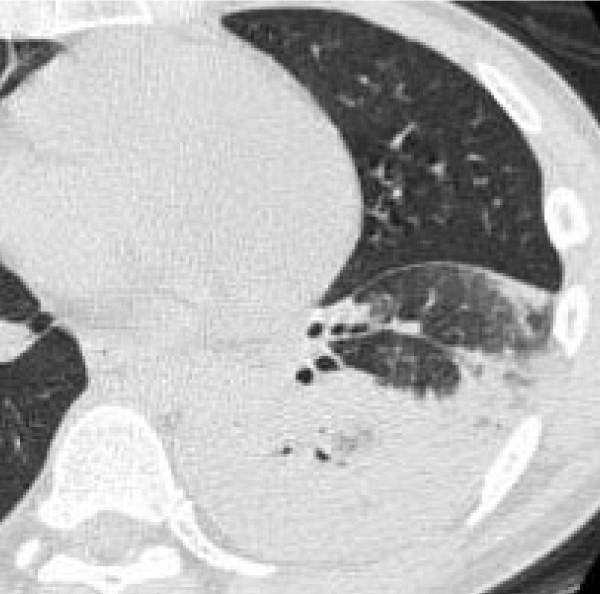
**39-year-old man with *S. pneumoniae *pneumonia**. CT shows air-space consolidation in left lower lobe.

Chest CT findings of the patients with *M. pneumoniae *pneumonia and *S. pneumoniae *pneumonia are listed in Table 2. Of the 64 patients with *M. pneumoniae *pneumonia, bronchial wall thickening was observed most frequently (81%), followed by centrilobular nodules (78%), ground-glass attenuation (78%), and consolidation (61%) (Figures 1 and 2). Reticular or linear opacities, pleural effusion, and lymphadenopathy were also observed, but at low frequencies. Among the CT findings, bronchial wall thickening and centrilobular nodules were observed more often in *M. pneumoniae *patients than in *S. pneumoniae *patients (*p *< 0.0001). Air-space consolidation was often seen in both pneumonias but as a non-specific finding (*p *= 0.0618) (Figure 3). Bronchial wall thickening or centrilobular nodules, which were considered to be a spectrum of HRCT findings for bronchopneumonia, were seen in 59 (92%) patients with *M. pneumoniae *pneumonia, and in 20 (29%) patients with *S. pneumoniae *pneumonia, which was a statistically significant difference (*p *< 0.0001). The presence of bilateral bronchial wall thickening or centrilobular nodules was only seen in patients with *M. pneumoniae *pneumonia.

**Table 2:** Chest CT findings of the patients with *Mycoplasma pneumoniae *pneumonia and *Streptococcus pneumoniae *pneumonia on admission*

Findings	*M. pneumoniae* (n = 64)	*S. pneumoniae* (n = 68)	*p *value
Consolidation	39 (61)	52 (76)	0.0618
Ground-glass attenuation	50 (78)	32 (47)	0.0003
Centrilobular nodules	50 (78)	15 (22)	<0.0001
Bronchial wall thickening	52 (81)	13 (19)	<0.0001
Reticular or linear opacity	17 (27)	22 (32)	0.5675
Pleural effusion	13 (20)	17 (25)	0.5409
Lymphadenopathy	15 (23)	13 (19)	0.6707

* Data represent the numbers of patients and numbers in parentheses are percentages.

The frequency of involvement in each lobe was not statistically different between patients with *M. pneumoniae *pneumonia and *S. pneumoniae *pneumonia (Table 3). However, the frequency of involvement of more than one lobe was seen more often in patients with *M. pneumoniae *pneumonia than in patients with *S. pneumoniae *pneumonia (*p *= 0.0005).

**Table 3:** Frequency of lobe involvement*

Parameter	*M. pneumoniae* (n = 64)	*S. pneumoniae* (n = 68)	*p *value
Lobe			
Upper	33 (55)	30 (44)	0.2960
Middle	31 (48)	22 (32)	0.0759
Lower	51 (80)	47 (69)	0.2319
More than one lobe involved	46 (72)	31 (47)	0.0005

* Data represent numbers of patients and numbers in parentheses are percentages.

Using the scoring system of the JRS guidelines, 12 of 64 patients with *M. pneumoniae *pneumonia were judged as bacterial pneumonia and 10 of 68 patients with *S. pneumoniae *pneumonia were judged as atypical pneumonia. Of the 12 patients with *M. pneumoniae *pneumonia who were judged as having bacterial pneumonia, 10 patients showed the radiographic features of *M. pneumoniae *pneumonia with the combination of bronchial wall thickening and centrilobular nodules. In contrast, 10 patients with *S. pneumoniae *pneumonia who were judged as atypical pneumonia did not show the radiographic features of *M. pneumoniae *pneumonia.

**Figure 4:**
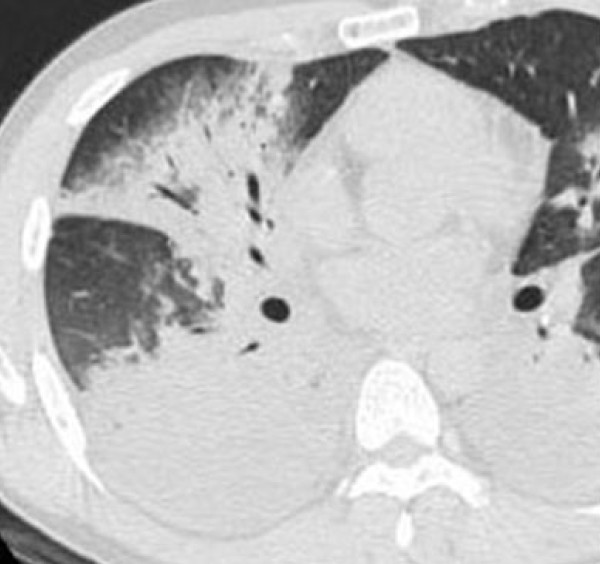
**31-year-old man with severe *M. pneumoniae *pneumonia with respiratory failure requiring mechanical ventilation**. CT shows bilateral air-space consolidation and pleural effusion.

During the study period, five of 64 patients with *M. pneumoniae *pneumonia progressed severe pneumonia with respiratory failure requiring mechanical ventilation (progressed stage). In these five patients, chest CT was performed in both the non-progressed (mild to moderate severity pneumonia without respiratory failure) and the progressed stage. The time between clinical onset of pneumonia (fever and/or respiratory symptoms) and CT (progressed stage) ranged from 8 to 15 days (mean, 10.9 days). All five patients showed the radiographic features of *M. pneumoniae *pneumonia, such as bronchial wall thickening and centrilobular nodules on admission. However, at the progressed stage these patients showed bilateral diffuse infiltrates and did not show the radiographic features of *M. pneumoniae *pneumonia that was observed in the non-progressed stage (Figure 4). There were no any differences in terms of clinical characteristics on admission between the five patients with progressed severe pneumonia and the remaining patients.

## Discussion

One of the basic policies and main purposes of the JRS CAP guidelines include the prevention of bacterial resistance because of the high frequency of drug-resistant respiratory pathogens among Asian countries [4]. As shown in Table 1, there are many clinical differences between *M. pneumoniae *pneumonia and *S. pneumoniae *pneumonia. These results were similar to those described in previous reports [16,19]. Thus, the JRS guidelines propose a differential diagnosis for atypical pneumonia, especially *M. pneumoniae *pneumonia, and bacterial pneumonia, predominantly *S. pneumoniae *pneumonia, for the selection of appropriate antibiotic management of CAP [4]. Usually, chest radiography is the first imaging technique obtained for the evaluation of acute respiratory symptoms. However, the patterns of presentation of *M. pneumoniae *pneumonia on chest radiography are nonspecific, consisting of patchy areas of airspace consolidation, reticular interstitial infiltrates, or both [10,19]. Thus, the JRS guidelines excluded chest radiographic findings from a differential table when the guidelines were updated [4]. In contrast, CT images can more accurately provide detailed information about the lung parenchyma than routine chest radiography. Reittner *et al*. compared the radiographic and chest CT findings of *M. pneumoniae *pneumonia and concluded that the lobular distribution, centrilobular involvement, and interstitial abnormalities in *M. pneumoniae *pneumonia are often difficult to recognize on radiographs but these features can usually be seen on CT [20].

In this study, the CT findings of *M. pneumoniae *pneumonia were evaluated and compared it with *S. pneumoniae *pneumonia, which is the leading pathogen of CAP worldwide. The most important differences between *M. pneumoniae *and *S. pneumoniae *pneumonias were the frequency of bronchial wall thickening and centrilobular nodules. These findings were observed more often in *M. pneumoniae *patients than in *S. pneumoniae *patients (*p *< 0.0001). Furthermore, the presence of bilateral bronchial wall thickening or centrilobular nodules may be highly suggestive for *M. pneumoniae *pneumonia. Nambu *et al*. reported that the difference in frequency of bronchovascular bundle thickening (bronchial wall thickening) and peribronchovascular or centrilobular nodules between *M. pneumoniae *and *S. pneumoniae *was statistically significant (*p *= 0.004) [15]. Air-space consolidation with or without centrilobular or peribronchovascular nodules were often seen in both pneumonias and were nonspecific. Reittner *et al*. also reported that centrilobular nodules were detected more frequently in *M. pneumoniae *pneumonia (86%) than in bacterial pneumonias (17%, *p *< 0.01) [20]. The finding of bronchial wall thickening was not evaluated in that study. These data together with the present results suggest that *M. pneumoniae *pneumonia may be differentiated from *S. pneumoniae *pneumonia using CT findings with the combination of bronchial wall thickening and centrilobular nodules.

To confirm whether CT findings can differentiate *M. pneumoniae *pneumonia from *S. pneumoniae *pneumonia, patients with *M. pneumoniae *pneumonia who were suspected as having bacterial pneumonia were evaluated by the scoring system of the JRS guidelines. Of these, 83% patients could be differentiated from *S. pneumoniae *pneumonia by radiographic features. In contrast, all patients with *S. pneumoniae *pneumonia who were suspected as atypical pneumonia did not show the radiographic features of *M. pneumoniae *pneumonia. Thus, the diagnosis of *M. pneumoniae *pneumonia appeared to be reliable when found with the combination of bronchial wall thickening and centrilobular nodules in the CT findings.

The *M. pneumoniae *organism attaches to cilia through the P1 protein and multiplies in the respiratory epithelial layer [1]. Attachment to epithelial cilia is essential for *M. pneumoniae *infection, through to progression and manifestation as bronchial wall thickening. Histopathologically, it is characterized by acute cellular bronchiolitis with edematous and ulcerative lesions of the bronchial walls and by peribronchial and perivascular interstitial opacities containing lymphocytes, plasma cells, and macrophages [21-23]. In contrast, *S. pneumoniae *may not have this biological capacity to directly infect the alveolar lumen, thereby allowing pneumonia to progress [24]. Although *S. pneumoniae *can occasionally damage the respiratory epithelium directly, it rarely produces ongoing disease in segmental bronchi. The main site of inflammation may reflect the CT findings.

Pneumonia due to *M. pneumoniae *is usually mild, but some cases are known to develop into severe, life-threatening pneumonia with respiratory failure and acute respiratory distress syndrome [18,25,26]. This study compared the CT findings between the early stage (mild to moderate severity) and progressed stage (severe pneumonia with respiratory failure) of *M. pneumoniae *pneumonia. Unfortunately, all patients with progressed pneumonia did not show the typical radiographic features of *M. pneumoniae *pneumonia. For further evaluation of CT findings at the progressed stage, 13 severe *M. pneumoniae *pneumonia with respiratory failure were evaluated that were described in a previous report [18]. These severe cases showed the same CT findings of the above progressed cases and could not be differentiated from *S. pneumoniae *pneumonia.

The limitation of the present study is the lack of inclusion of pneumonia due to other common microorganisms such as *C. pneumoniae *or *Haemophilus influenzae*. Because *C. pneumoniae *pneumonia is usually mild and self-limiting, it is treated in an outpatient setting without CT scanning [3]. *H. influenzae *is usually detected from patients with chronic lung diseases such as bronchiectasis, diffuse panbronchiolitis, or chronic obstructive pulmonary disease. Thus, it is difficult to evaluate the precise CT findings in *H. influenzae *pneumonia. Another limitation of the present study is that it excluded patients with chronic lung diseases and severe pneumonia for the precise evaluation of CT findings. These diseases could influence CT findings. In addition, *M. pneumoniae *usually infects young adults with no or mild co-morbid illnesses [16,18,19]. A further weakness of this study is its retrospective design and small sample size, which are characteristic of such preliminary studies. Thus, further studies are necessary to determine whether CT findings could differentiate the various microorganisms with or without chronic lung diseases. However, the usefulness of CT findings is limited to hospitals with CT scanners. The guidelines are aimed at general practitioners and non-specialized doctors and were selected to allow easy differentiation of CAP in an outpatient setting without special examinations [4]. Thus, the JRS guidelines may not recommend the utilization of chest CT routinely.

## Conclusion

The main purpose of the present study was to identify a means of rapidly distinguishing *M. pneumoniae *pneumonia from *S. pneumoniae *pneumonia in daily clinical practice without waiting for serological results. The results indicate that the combination of bronchial wall thickening and centrilobular nodules on chest CT is most suggestive of *M. pneumoniae *pneumonia. The presence of bilateral bronchial wall thickening or centrilobular nodules was highly suggestive of *M. pneumoniae *pneumonia.

## Competing interests

The authors declare that they have no competing interests.

## Authors' contributions

NM and KO conceived the study and participated in its design and coordination. NM, TS, KO, YK, and MO collected and managed the data, including quality control. YK, KO, and TY carried out the microbiological laboratory tests. NM and KO drafted the manuscript, and all authors contributed substantially to its revision. All the authors read and approved the final manuscript.

## Pre-publication history

The pre-publication history for this paper can be accessed here:

http://www.biomedcentral.com/1471-2342/9/7/prepub
